# Ga-Based Alloys in Microelectronic Interconnects: A Review

**DOI:** 10.3390/ma11081384

**Published:** 2018-08-08

**Authors:** Shiqian Liu, Keith Sweatman, Stuart McDonald, Kazuhiro Nogita

**Affiliations:** 1 Nihon Superior Centre for the Manufacture of Electronic Materials (NS CMEM), School of Mechanical and Mining Engineering, The University of Queensland, Brisbane 4072, QLD, Australia; s.mcdonald1@uq.edu.au (S.M.); k.nogita@uq.edu.au (K.N.); 2 Nihon Superior Co., Ltd, Suita City, Osaka 564-0063, Japan; k.sweatman@nihonsuperior.co.jp

**Keywords:** Ga-based alloy, room temperature liquid alloy, microelectronic interconnect

## Abstract

Gallium (Ga) and some of its alloys have a range of properties that make them an attractive option for microelectronic interconnects, including low melting point, non-toxicity, and the ability to wet without fluxing most materials—including oxides—found in microelectronics. Some of these properties result from their ability to form stable high melting temperature solid solutions and intermetallic compounds with other metals, such as copper, nickel, and aluminium. Ga and Ga-based alloys have already received significant attention in the scientific literature given their potential for use in the liquid state. Their potential for enabling the miniaturisation and deformability of microelectronic devices has also been demonstrated. The low process temperatures, made possible by their low melting points, produce significant energy savings. However, there are still some issues that need to be addressed before their potential can be fully realised. Characterising Ga and Ga-based alloys, and their reactions with materials commonly used in the microelectronic industry, are thus a priority for the electronics industry. This review provides a summary of research related to the applications and characterisation of Ga-based alloys. If the potential of Ga-based alloys for low temperature bonding in microelectronics manufacturing is to be realised, more work needs to be done on their interactions with the wide range of substrate materials now being used in electronic circuitry.

## 1. Introduction

A noteworthy trend is being observed with microelectronic products becoming smaller and more energy efficient [1,2], and performing well while having minimal impact on human health and the environment [3]. Also, both academic and industrial interest has been expressed in flexible or deformable electronics. To meet these challenges, new technologies for manufacturing advanced microelectronic components and packaging of electronic devices are required.

Alloys that are liquid at room temperature or that have a low melting point have the potential to create unique combinations of electrical and thermal conductivity. They offer the additional benefit of enabling the production of flexible electronic circuitry. Much research has been completed on the microelectronic components and circuit innovations that use liquid metals, and the efforts are continuing. These alloys also have the potential of enabling electronic assembly at lower process temperatures than those required by conventional alloys. As well as saving energy, low process temperatures allow the assembly of components and materials that can be damaged by the process temperatures required for assembly with conventional solder alloys, such as the traditional Sn-37Pb solder with a melting point of 183 °C and the lead-free solders required by the European Union’s Restriction for Hazardous Substances (RoHS) regulations, such as Sn-3.8Ag-0.7Cu with a melting point of 217 °C [4].

Several metals and alloys have melting points near room temperature. However, issues such as radioactivity (Cs and Fr), short half-life (Fr), and violent chemical properties (Cs and Rb) limit their applications in microelectronics. Though widely used in microelectronics, Hg, Pb, and their alloys are toxic and their use in electronics is limited by regulations such as RoHS [4].

With a melting point of 29.76 °C and a boiling point of 2403 °C [5,6], Ga has a large temperature range for liquid state applications. Some Ga-based alloys have even lower melting points. The eutectic Ga-In alloy (EGaIn, 75.5 wt % Ga and 24.5 wt % In) has a melting point 15.5 °C [7]) and Galinstan (a commercial eutectic Ga-In-Sn alloy from Geratherm Medical AG in Germany and distributed by RG Medical Diagnostics in the U.S., 68.5 wt % Ga, 21.5 wt % In, and 10.0 wt % Sn) has a melting point of −19 °C [8]. Since Ga is considered to be non-toxic and environmentally friendly [6,9], Ga and Ga-based alloys appear to be a promising substitute for toxic metals in a range of liquid metal applications.

This review provides a summary of research related to the characterisation of Ga-based alloys and their applications in microelectronics.

## 2. Properties of Ga and Ga-Based Alloys

Physical properties of Ga, EGaIn, and Galinstan are shown in Table 1. Ga-based alloys have similar properties to Ga. Data for Hg are listed as a comparison.

Ga has a low vapour pressure at high temperatures, especially when compared to mercury, which is currently the most commonly used room temperature liquid metal. As well as its low toxicity, its low vapour pressure also makes it a safe alternative to Hg and Pb. Liquid Ga can wet many kinds of materials, including most metals and glasses. It has low viscosity and good conductivity (Table 1). Ga exhibits significant undercooling on solidification [13]. It expands by 3.1% on solidification, which is different from most other metals. The thermal expansion coefficient of Ga (18.3 × 10^−6^ K^−1^) is closer to that of Cu (16.7 × 10^−6^ K^−1^) than Sn (20.5 × 10^−6^ K^−1^) or Pb (29.0 × 10^−6^ K^−1^) at 20 °C [9]. When contained in flexible tubing at room temperature, liquid Ga-based alloys have been shown to flow and maintain metallic conductivity while being stretched [14].

**Figure 1:**
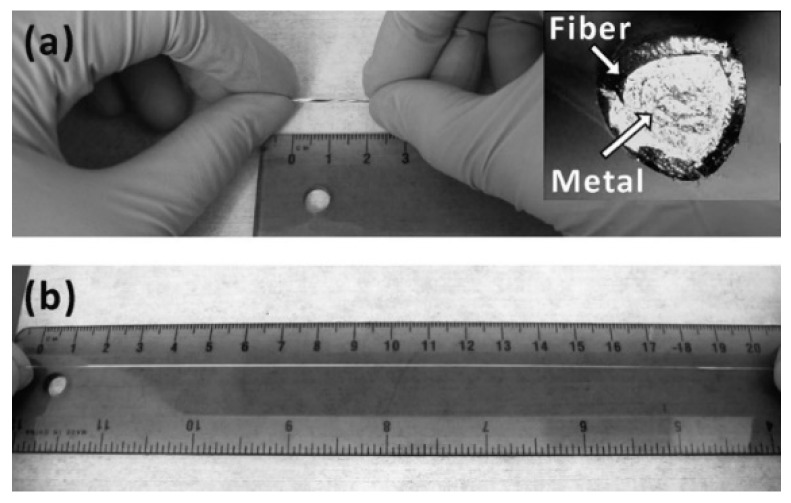
(**a**) A relaxed, 2 cm section of a stretchable conductive fibre. The shiny core of its cross-section (inset) is the liquid metal. (**b**) The fiber is stretched to 20 cm and the metal appears to uniformly fill the stretched fiber [14].

**Figure 2:**
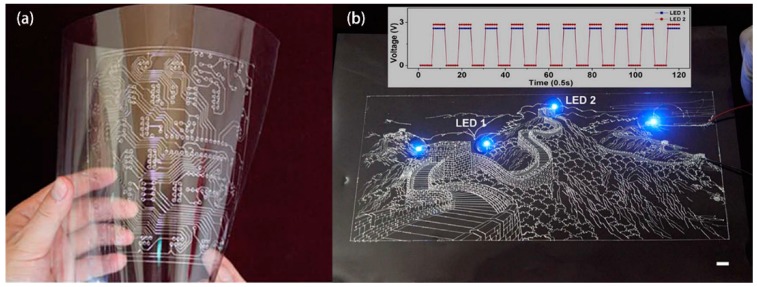
Direct-printed functional electronic patterns composed of liquid metal wires on a polyvinyl chloride (PVC) substrate for integrated circuit (IC) application: (**a**) the printed circuit board (PCB) that can be folded; (**b**) the “Great Wall” pattern with illuminated light-emitting diodes (LEDs). Reproduced from Zheng et al. [57] under Creative Commons Attribution-NonCommercial-ShareAlike 3.0 Unported license.

Although semiconductor applications such as GaAs and GaN dominate the commercial demand for Ga [6,15,16,17], Ga and Ga-based alloys have attracted significant attention as possible replacements for liquid metals in various applications because of their remarkable fluidity and metallic properties at low temperatures, as well as their lack of toxicity. Ga and its alloys have been proposed as replacements for Hg in dental filling materials [18,19,20] and high-temperature thermometers [21,22,23,24]. Ga and Ga-based alloys have also been studied as potential coolants in nuclear power plants as an alternative to Na [10,25,26,27,28,29], and in microelectronic devices, such as computers and smart phones [30,31,32,33,34,35]. Eutectic Ga-In (EGaIn) alloys and Ga-In-Sn alloys (such as Galinstan) have received the most attention in the literature in terms of microelectronic applications. The inherent properties of Ga and Ga-based alloys enable their use in microelectronics where deformability, miniaturisation, low process temperature during fabrication, and low toxicity are required. The growing demand for wearable devices and artificial body parts, including prosthetics and implantable device, has inspired research and the production of deformable electronics [36,37]. Many exciting and promising circuits [14,38,39,40,41,42] and electronic components [43,44,45,46,47,48,49,50,51] that can be bent (flexible) or elongated (stretchable) have been developed in recent years, although these applications are mainly at the laboratory stage. Figure 1 and Figure 2 are examples of flexible or stretchable applications based on Ga alloys. In order to realise these potential applications, basic processing techniques with liquid Ga based alloys have been studied [52]. These efforts include injecting [7] and printing [53,54,55,56] the liquid alloy into or onto various microfluidic channels or substrates. The substrates tested are mainly polymers, such as polydimethylsiloxane (PDMS) and polyvinyl chloride (PVC). Liquid metal patterning is emerging as a major area in room temperature liquid metal research.

## 3. Ga and Ga-Based Alloys Applications in Microelectronic Interconnects

### 3.1. Soldering

Soldering has been a fundamental interconnection technology in microelectronic packaging since the commencement of the electronic age. During the soldering process, the gap between the surfaces to be joined (usually metals) is filled with a molten alloy that has the ability to wet the substrates, usually with the assistance of a flux that removes oxides from the substrates and the molten alloy. Traditional Sn-37Pb solder has a melting point of 183 °C. Pb-free solders that are now widely used in electronic assembly have even higher melting points. Sn-3Cu-0.5Cu (“SAC305”), has a melting range of 217–220 °C and Sn-0.7Cu-0.05Ni (SN100C^®^), has a melting point of 227 °C. The temperature required in processes based on those solders has the potential to damage the heat-sensitive materials used in electronic devices, including modern microchip components and flexible substrates such as polyester film. There have been ongoing efforts to study various alloying elements including Ag, Bi, Cd, Cu, In, Sb, Zn, and Al to create alloys that make possible low temperature soldering processes [58], to reduce energy consumption, and to reduce the risk of damaging components. Their low melting points, together with the possibility of forming intermetallic compounds (IMCs) with other metals meant that Ga and Ga-based alloys are potential joining materials in microelectronics.

**Figure 3:**
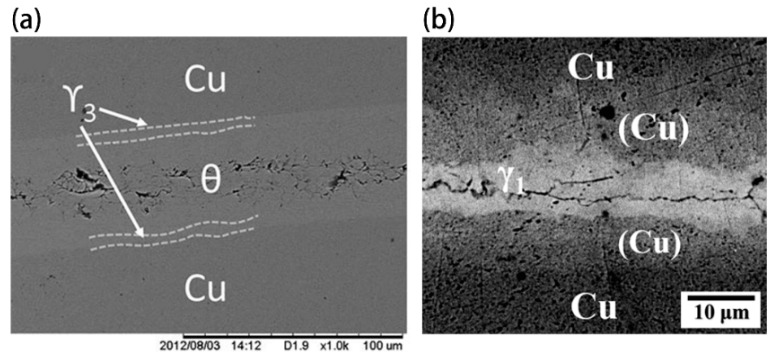
The backscattered images of (**a**) a Cu/Ga/Cu sandwich couple produced after 96 h at 160 °C [60]; (**b**) a Cu/Pt/Ga/Pt/Cu couple produced at 300 °C for 24 h [59], where γ_1_ and γ_3_ are γ_1_-Cu_9_Ga_4_ and γ_3_-Cu_9_Ga_4_, respectively.

The feasibility of using pure Ga paste to realise transient liquid phase bonding with a Cu substrate (with and without Pt coating) at processing temperatures ranging from 160 to 300 °C has been examined [59,60]. In the studies, Cu/Ga/Cu and Cu/Pt/Ga/Pt/Ga sandwich couples have been fabricated at 160 and 300 °C, respectively, to gain knowledge for developing practical industrial Ga-based solder materials (Figure 3).

**Figure 4:**
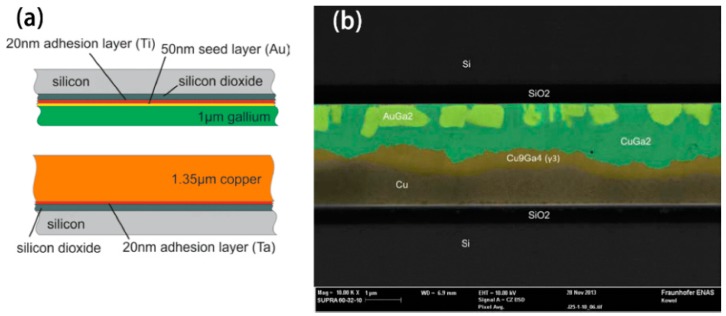
Low-temperature wafer bonding using solid-liquid inter-diffusion of Ga/Cu system [61]. (**a**) Schematic cross section of the interface prepared for the Ga/Cu bonding; (**b**) scanning electron microscopy (SEM) cross section of Ga/Cu bonded samples after annealing at 90 °C.

The bonding between a magnetron sputtered Cu layer and an electroplated Ga layer on a 50 nm Au seed layer has been investigated [61]. A schematic cross section of the interface to be joined is illustrated in Figure 4a. The bonding of these samples was performed at 25 °C in commercial wafer bonding equipment with a pressure of 2.5 MPa and holding time of 10 min. Subsequently, the wafers were annealed at different temperatures up to 200 °C for 80 h. After annealing at 90 °C, a shear strength of up to 90 MPa was achieved. The scanning electron microscopy (SEM) image of the bond cross section is shown in Figure 4b.

In addition to the cases in which pure Ga was used as a joint material, some Ga-based alloys have also been studied.

A ternary Sn-32 wt % Bi-6 wt % Ga alloy with a melting point of 128 °C was developed as a solder alloy [62]. The Ga content in this alloy is the maximum amount possible without forming a liquid phase or creating the possibility of Ga segregation. This new solder was reflowed on Cu substrates at 158 °C and then annealed at 70, 90, and 110 °C for 24–720 h to determine the IMCs that formed and the evolution of the interfacial morphology as a function of time. The only IMC phase observed at the interface area was CuGa_2_. The shear strength of the joints formed by reflowing this solder alloy on Cu substrate with an organic solderability preservative (OSP) coating was measured and the authors concluded that although the CuGa_2_ IMC is brittle, this solder is still a potential candidate for low-temperature microelectronics packaging.

As well as the Sn-Bi-Ga alloy, a eutectic Ga-Zn (Ga-10 wt % Zn, melting point 24.7 °C) solder paste has been proposed, and the interactions with single-phase brasses at 150 and 200 °C have been investigated during the soldering process [63]. This Zn-containing Ga alloy has advantages in both the manufacturing process and joint quality, and is regarded as a promising material for low-temperature diffusion soldering of materials used in microelectronics.

The solderability of the eutectic Ga-Sn (EGaSn, Ga-13.5 wt % Sn, melting point 20.5 °C) liquid alloy on Au-coated Cu substrates was observed by forming sandwich joints and holding them at room temperature or 100 °C for seven days [64]. Pressure (10 kPa) was required during the process in order to form the bond. This study showed the possibility of using EGaSn in low temperature bonding of Cu substrates.

The bonding of Ga and Ga-based alloys with substrates other than Cu has also been investigated. A composite soldering paste for cermet sections has been developed using Ga as the low-melting base and a two-part powder mixture (Cu-20 wt % Sn alloy, and eutectic Ag-28.1 wt % Cu alloy) as the high temperature filler [65]. Several materials, including titanium wrought alloy (OT4-VK94-1), aluminum alloy (M1-SK-1) and bronze (BrB2-SK-1), were soldered using this paste. This solder needs a process temperature of 200 °C, a holding time of two hours and a pressure of 3-4 MPa to make a joint with adequate strength.

**Figure 5:**
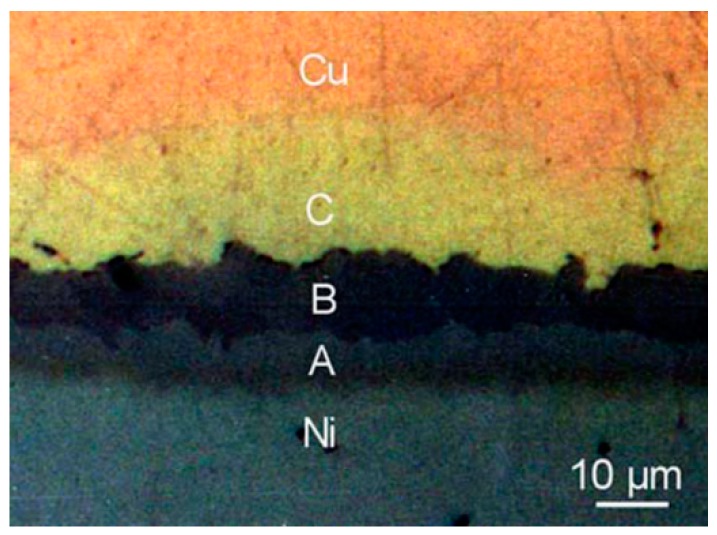
Optical microscope image of the Cu/Al-Ga-Ni paste/Ni bond cross section [66]. A, B, and C are α’-Ni_3_Ga, β-Cu_3_Ga, and Cu solid solution phase, respectively.

A metallic paste created by mixing Ga, Al, and Ni powders (45 wt % Ga, 15 wt % Al, and 40 wt % Ni) has been reported [66]. The diffusion soldering process was conducted at a temperature of 700 °C for 20 min. Pb-free joints were produced between Cu and Ni substrates by forming a solid solution and IMCs that are stable even at temperatures of about 1200 °C. As shown in Figure 5, the phases detected in the joint interface area were: α’-Ni_3_Ga (labelled ‘A’ in the image), β-Cu_3_Ga (‘B’) and a Cu solid solution (‘C’) of the Ga-Cu system.

The flip-chip interconnect applications of Ga-based alloys have also been studied [67]. Ga-Cu-Ni ternary alloys (Ga-30 wt % Cu-5 wt % Ni and Ga-21.4 wt % Cu-3.6 wt % Ni) have been used as a micro-miniature interconnect to join bare silicon chips and printed circuit boards. The bonding process began with pressure being applied for one hour, after which the test vehicles were placed in a convection oven at 150 °C for one hour to complete the Ga alloy and underfill cure.

The processability of Ga, 30 wt % Cu, 5 wt % Ni alloy for room temperature via filling applications has also been demonstrated using stencil printing onto stainless steel substrates [68]. After filling, a 16 h cure at 130 °C was required to achieve interconnections. The research results suggest that this alloy has potential as a low temperature interconnect material.

### 3.2. Heat-Free Bonding

Stable undercooled liquid nano- and/or micro-particles have been considered for use as a low temperature solder [69]. Since Ga and some of its alloys are liquid near or at room temperature and exhibit significant undercooling on solidification, a few attempts have been made to eliminate the heat required in conventional soldering.

**Figure 6:**
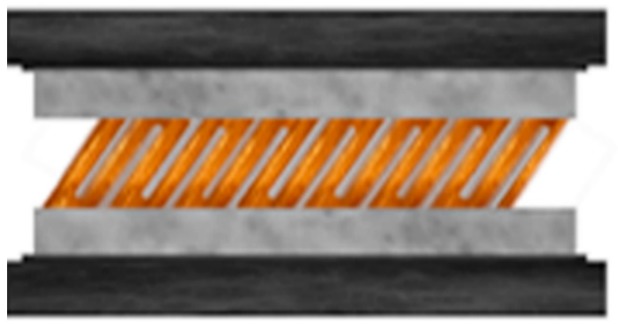
Low-temperature metallic gluing enabled by Ga- and In-coated nanorods [70].

A new kind of metallic glue termed “MesoGlue Eutectic” has been developed [70]. The bond is achieved by first planting Ga and In coated rods alternately along a substrate. The teeth-on-comb rods on each substrate are interlaced. When they contact the In and Ga form a liquid. The eutectic liquid eventually turns into a solid and forms a metal bond. The gluing can be performed at room temperature and in air, although it requires some pressure (<100 psi). Figure 6 is a schematic diagram of this metallic gluing bond. The bond is about as strong as a metallurgical bond between two parent materials. This metallic glue is proposed for use as a joining method in the microelectronic packaging industry, and may replace thermal greases as it transfers heat more efficiently. At this point, this metallic glue has only been applied in a laboratory.

Another type of metallic cement (or metallic glue) is obtained by the chemical interaction of liquid metals with metallic alloy powders. This type of cement has high adhesion to materials of different types and presents good heat and electric conductivity. This type of bond can be widely applied to joining ceramics, metals, quartz, graphite, and other thermostable materials in aviation and electronics industries and in instrument engineering. Grigor’eva et al. [71] investigated the structure of metallic cements formed by the interaction of Cu/Bi mechanocomposites with liquid Ga at room temperature. The Cu/Bi mechanocomposites are mechanically activated powders obtained by mixing Cu and Bi powders in planetary ball mill in an argon atmosphere. The application of the Cu-Bi mechanocomposite, instead of Cu, as a filler for obtaining Ga-based glues not only reduces the curing time of the cement from two days to 14 h, but also increases the compressive strength of joints from 30 to 70 MPa.

Ye et al. [72] took advantage of the low melting temperature of Ga and found that Ga layers can act as reversible and switchable adhesives. The adhesion status can be controlled by slight temperature changes. The switch is sensitive and the joint has a similar strength to a conventional glue. The temporary adhesion is electrically conductive, repeatable, and leaves the surface clean after lifting. Applications of this adhesion can be found in areas such as industrial pick-and-place processes and temporary wafer bonding. Adhesive switchability is proposed as an enabling technology for the feet of climbing robots.

## 4. Characterisation of Reactions between Liquid Ga-Based Alloys and Solid Metals

Various characteristics of pure liquid Ga and liquid Ga-based alloys (mainly EGaIn and Galinstan) have been observed. These includes basic thermophysical [11,21,34,44,45,73,74], electrochemical [46,75,76,77], electromagnetic [78,79], fluidity and wettability properties [80,81,82], and self-fueled actuation, in which chemical energy spontaneously converts into mechanical activity to induce autonomous locomotion [77,83,84]. These research achievements lay the foundation for some techniques for liquid alloy control and utilisation, such as patterning the deposit liquid metal [7,85,86] and fabricating small liquid metal particles [87,88,89]. These techniques further enable a variety of promising applications as described in the previous chapter.

Notably, the interfacial reactions between the liquid Ga-based alloy and solid materials are important for making better use of these materials, since liquid alloy/solid state contact is common in all the applications mentioned in this study. More specifically, the interfacial reactions could be divided into microstructure development in the interfacial layer and wettability. In some situations, these two processes can influence one another, so understanding the mechanisms in these two aspects can be complex. These fundamental but important research achievements help in the selection of reliable materials for these applications and thus enable the further use of Ga and Ga-based alloys.

### 4.1. Microstructure Development between Liquid Ga-Based Alloy and Solid Materials

To understand microstructure development, the reaction products between the liquid and solid layers need to be identified and their morphologies observed. These affect the contact quality and stability as measured by thermal and electrical conductivity and/or joint strength.

During the interaction between the liquid Ga alloy and metal substrate or powder, interfacial IMCs usually form. Thus, dissolution of the materials, IMCs composition, epitaxial growth of the IMC grains, formation of solid/liquid or even new solid/solid interfaces, and their evolution with time or surrounding environment (temperature, atmosphere, etc.) may all require consideration.

#### 4.1.1. Liquid Ga-Based Alloy Reactions with Metal Powder

The interaction between liquid Ga (or Ga-based alloys) and Cu or single phase Cu alloy powder has been the focus of several research papers [90,91,92,93,94,95,96]. The researchers mainly used Cu, Cu-based solid solution, or IMC powders and studied their reaction with liquid Ga or liquid Ga-based alloy, and investigated the formation of IMCs. This group of researchers used the mechanochemical method (planetary ball-milling in an argon atmosphere) to obtain the solid solution or IMC powder, which was then mixed with the liquid Ga (or Ga-based alloys). The in-situ X-ray diffraction (XRD) observations were recorded at the Siberian Center of Synchrotron Radiation, Russia. Their results showed that, when pure Cu powder, Cu + 20 wt % Ga solid solution, or Cu_9_Ga_4_, are mixed with liquid Ga in proportions approximately corresponding to the stoichiometry of the main reaction of Cu with Ga at a small Cu excess, CuGa_2_ is the single product of the interaction at room temperature. In the Cu solid solution (with Sn, In, or Bi) Ga liquid eutectic systems, the first phase to form is a microcrystalline IMC. Then, after an induction period, the IMC resulting from the reaction between Cu and Ga appears. The metallic phase consists of much coarser grains in comparison with the starting powder and primary (IMC) phase. All the reactions are summarized in Table 2.

The increasing Sn concentration in the Cu-Sn solid with liquid Ga or EGaSn system shortens the induction period for the crystallisation of the Sn phase. For Cu(Sn) powders reacting with liquid Ga, Sn segregation into an autonomous phase occurred 30 h after the components were mixed. When using EGaSn as the liquid phase, Sn was segregated into an autonomous phase four hours after the components were mixed. When using mechanochemical synthesized Cu_3_Sn or Cu_6_Sn_5_, CuGa_2_ and Sn appeared almost immediately after mixing, and all Ga from the eutectic melt was consumed by CuGa_2_ formation in 10 and 2 h, respectively. The IMC phase decreased in size as the Sn concentration increased. For the Cu_3_Sn + EGaSn, the IMC CuGa_2_ had particle sizes of at most 1 μm; Sn had far coarser particles. For the Cu_6_Sn_5_ + EGaSn, the CuGa_2_ crystal sizes were less than 0.1 μm.

Besides the characterisation of the solid solution powder/ liquid Ga system for metallic bonding material fabrication, research on another series of Ag-Sn-Cu-based alloy powders mixed with Ga-In-Sn liquid has been completed for dental use [20,97,98]. Several techniques were employed to investigate the reaction mechanisms during the setting process and the microstructural morphology evolution of these dental alloys, including SEM, XRD [97], transmission electron microscopy (TEM) [98], and differential scanning calorimetry (DSC) [20]. When these Ag-Sn-Cu-based alloy powders are mixed with the Ga-In-Sn liquid at room temperature, the materials transform to a solid. θ-CuGa_2_, Ga_28_Ag_72_, β-Sn, Ag_3_Sn, γ-Cu_9_Ga_4_, Ag_9_In_4_, and hexagonal Ag_2_Ga were reported to be observed in the system, as listed in Table 2 [99]. The reaction phase composition and the setting reaction kinetics influenced the clinically relevant properties, including the mechanical strength, corrosion resistance, and biocompatibility of these dental amalgams [99].

#### 4.1.2. Reactions between Liquid Ga-Based Alloys and Cu Substrates

When using these alloys in microelectronics, liquid Ga-based alloys and solid substrates interact.

Since Ga has a high solubility in the face centered cubic (FCC) Cu-rich phase, and there are several IMCs that could form over a large temperature range [100]. Ga-based solders attract attention when the materials for connecting Cu substrates in microelectronic packaging have to be chosen.

##### Microstructure Evolution

Bulk Cu/Ga couples and Cu/Ga/Cu sandwich couples with reactions at moderate processing temperatures ranging from 160 to 300 °C have been studied [59,60]. Room-temperature IMC formation and the relevant interdiffusion behaviour in thin-film Cu/Ga [101] couples have been analysed. Reaction conditions and products are summarised in Table 3.

**Figure 7:**
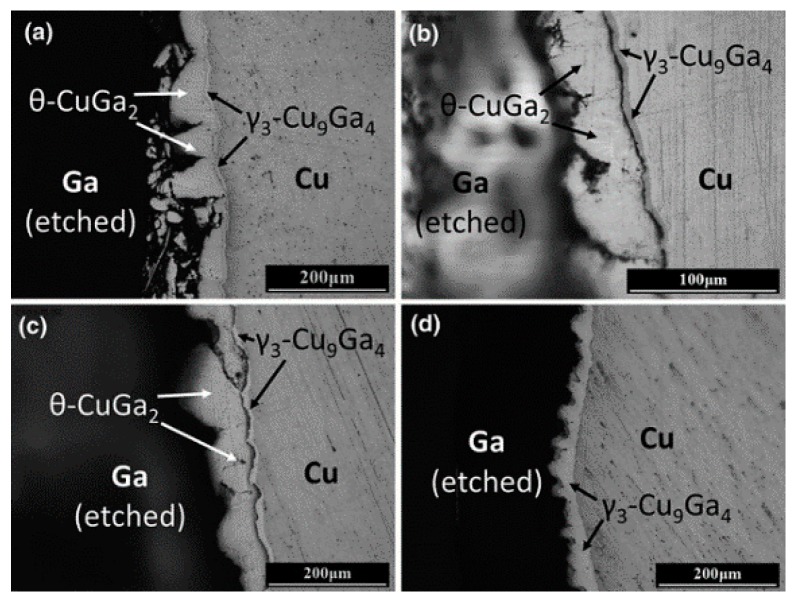
Ga/Cu couples reacted at 200 °C for (**a**) 3 h, (**b**) 6 h, (**c**) 24 h, and (**d**) 48 h [60].

**Figure 8:**
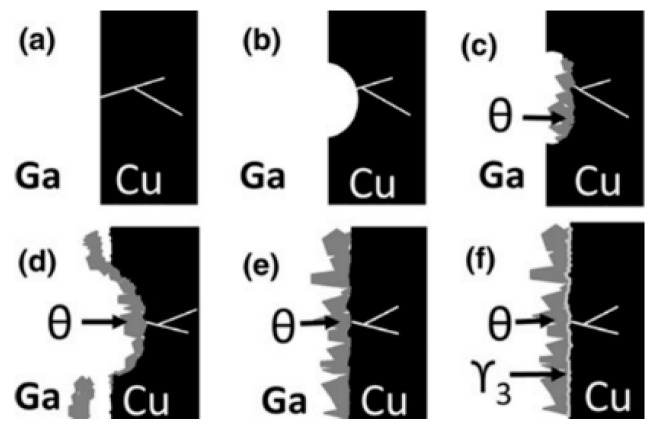
Schematic representation of the microstructural evolution of Ga/Cu [60]. (**a**)–(**f**) show the evolution in chronological sequence.

Figure 7 shows the interfacial microstructure of Ga/Cu couples reacted at 200 °C from 3 to 48 h. Diagrammatic sketches in Figure 8 interpret the microstructure evolution when liquid Ga is exposed to Cu substrates (Figure 8a), Cu dissolves into the liquid at certain localised sites and forms a “basin-type” depression (Figure 8b). Then, the θ-CuGa_2_ phase nucleates at sites within the basins (Figure 8c). As the reaction progresses, the θ-CuGa_2_ phase is also found in the remaining uncorroded areas (Figure 8d). During this stage, a very thin layer of γ_3_-Cu_9_Ga_4_ phase can be detected in the basins/depressions, between the θ-CuGa_2_ crystals and the Cu substrate. As the reaction time increases, a planar IMC layer is formed and thickens, and extends to cover the substrate (Figure 8e). Finally, the γ_3_-Cu_9_Ga_4_ phase thickens and forms a continuous layer that covers all the interface between the Cu and the CuGa_2_ (Figure 8f). As a result of substrate dissolution in liquid Ga and consumption in IMC growth (also referred to as a type of erosion), the Cu interface retreats.

**Figure 9:**
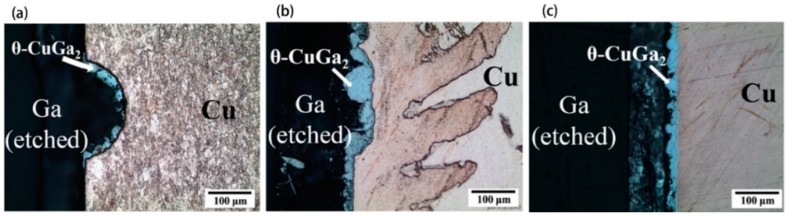
The optical microscope images of (**a**) Ga/polycrystalline-Cu couple reacted at 200 °C for 3 h, (**b**) Ga/single-crystalline-Cu couple reacted at 200 °C for 24 h, and (**c**) Ga/Pt/polycrystalline-Cu couple reacted at 200 °C for 6 h [59].

The basin-type morphology in the interface is caused by non-uniform reactions and is referred to as liquid metal embrittlement. This kind of interface has a negative effect on the joint strength and efforts have been made to inhibit this process. Methods that have been tried include changing the substrate from polycrystalline to a single-crystal to eliminate grain boundary effects [59], improving the wettability of liquid Ga on substrates by the addition of interlayers [59], and controlling the amount of liquid Ga [60]. The results in Figure 9 show that the non-uniform morphology forms at both the Ga/polycrystalline Cu and the Ga/single-crystal Cu interface, though the degree of “basin” formation in the Ga/polycrystalline Cu interface is greater. This suggests that grain boundaries are not the only reason for the basin-type interface formation. By sputtering Pt under bump metallization on polycrystalline Cu substrates, the wettability of the Cu substrate improved and the IMC layer grew uniformly along the interface.

**Figure 10:**
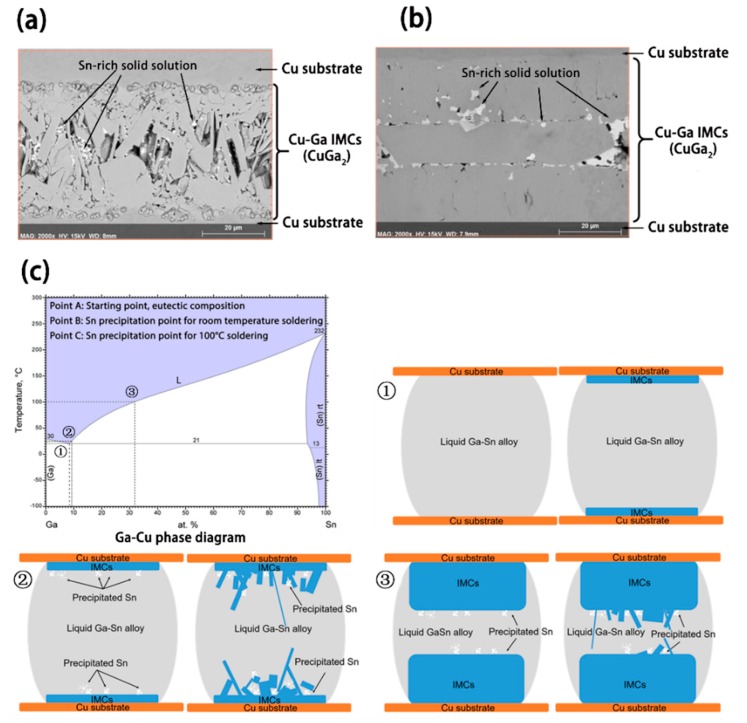
Microstructure evolution of joints formed between EGaSn and Au-coated Cu substrates [64]. (**a**) SEM image of a Cu/Au/EGaSn/Au/Cu substrate sandwich couple formed at room temperature; (**b**) SEM image of a Cu/Au/EGaSn/Au/Cu substrate sandwich couple formed at 100 °C; (**c**) schematic diagram of sandwich joint interface formation showing the relative difference in the intermetallic compound (IMC) thickness at the time of Sn precipitation at room temperature (**c**-**②**) and at 100 °C (**c**-**③**). At the starting point (**①**), liquid GaSn alloy contained 91.6 at% Ga and 8.4 at% Sn. The continuous planar IMC layer grows, which causes the Ga concentration in the liquid to drop. For the room temperature joint, the Ga concentration quickly reaches the limit where solid Sn (Ga) starts to form (**②**). At this point, the independent growth of Sn (Ga) disrupts the growth of planar IMCs. For the sandwich couple at 100 °C, solid Sn (Ga) will not form until the concentration of Ga in the liquid GaSn alloy decreases to 68.2 at% (**③**). Compared with the reactions at room temperature, the solid Sn (Ga) precipitation occurs much later at 100 °C.

There are drawbacks to these low temperature bonding methods using Ga and Ga-based alloys. A lack of hermeticity is one of the common problems that occurs during the bonding process as a consequence of the microstructure evolution, shown in Figure 3. Notably, cracks occur in the θ-CuGa_2_ phase regions of the Cu/Ga/Cu joints [59,60]. Similar cracks were found within the γ_1_-Cu_9_Ga_4_ phase in the Cu/Pt/Ga/Pt/Ga joints [59] and the Cu/Au/EGaSn/Au/Cu joints [64], as shown in Figure 10a,b.

One explanation for the cracks and voids in the Cu/Ga/Cu sandwich couples is the brittle nature of the θ-CuGa_2_ phase formed at the joint interface. Based on this possibility, several methods have been proposed to improve the joint reliability. One such method involves using a higher bonding temperature to mitigate the formation of the θ-CuGa_2_ phase. Froemel et al. [61] found that by increasing the annealing temperature (from 90 to 200 °C), the amount of CuGa_2_ decreased, favouring the formation of Cu_9_Ga_4_, and the shear strength increased correspondingly. Another hypothesis considered that the cracks were formed during the liquid-solid reaction instead of the physical vibration [64]. The different morphologies of the Cu/Au/EGaSn/Au/Cu joint interface are likely to arise from different temperatures of Sn precipitation, as demonstrated in Figure 10c. The two layers of Sn shown in Figure 10b demarcate the liquid/solid interface location when the Sn(Ga) precipitation occurred.

Another kind of defect formed in the interface are Kirkendall voids, which are caused by the fast diffusion of the substrate materials into the molten Ga alloy [61,102]. Thus, the interdiffusion coefficient of the liquid/solid couple is an important consideration when selecting the joining alloy.

##### IMC Properties

As can be seen from the Cu-Ga phase diagram and microstructure analysis, the main IMCs formed in the Cu-Ga system at low temperature are CuGa_2_ and Cu_9_Ga_4_. A study showed that effective packaging of EGaIn with CuGa_2_ would remarkably enhance the electrical conductivity (6 × 10^6^ S m^−1^, ∼80% increase) and thermal conductivity (50 W m^−1^ K^−1^, ∼100% increase) compared to EGaIn [103]. The result further indicated the possibility of utilizing Ga-based alloys in microelectronic interconnections to provide good thermal and electrical conductivity as well as mechanical support.

The thermal stability of the CuGa_2_ phase was studied in an inert atmosphere [104]. In this study, the authors found that the CuGa_2_ transforms to Cu_9_Ga_4_ and liquid Ga at 258 °C, rather than the 225 °C predicted from modelling. Thermal diffusivity and thermal conductivity of CuGa_2_ at temperatures between 25 and 227 °C were measured.

Of the commonly used joining materials listed in Table 4, the Cu/Ga system shows the smallest volume change. This means that the strain introduced by the reaction that occurs during the bonding process is the smallest, which could have implications for subsequent reliability of the joint.

θ-CuGa_2_ phase has been shown to be brittle when compared to the γ_1_-Cu_9_Ga_4_ phase [61]. The CuGa_2_ phase exists at low temperatures and is converted to Cu_9_Ga_4_ as the temperature increases. The mechanical strength of the bond increases and the electrical resistance decreases as the proportion of CuGa_2_ decreases, whereas that of Cu_9_Ga_4_ increases [61].

#### 4.1.3. Reactions between Liquid Ga-Based Alloys and Other Substrates

Reactions between Ga-based alloys and substrates other than Cu are less well characterized than those with Cu substrates. The dissolution and diffusion-reaction processes between Ga pastes (Ga-40 wt % Ni-15 wt % Al, or Ga-45 wt % Al) and two substrates (Cu and Ni) at 700 °C were analysed [66]. The IMCs and solid solution layers formed at the interface are shown in Figure 4. Room-temperature IMC formation and the relevant interdiffusion behavior in thin film Au/Ga [105] and Pd/Ga [101] couples has been analysed, and Au has been identified as a rapidly diffusing species. The suitability of Ga-In-Sn alloy solder as a detachable contact material with thermoelectric materials CoSb_3_, Mg_2_Si, and FeSi_2_ has been studied [106]. W, Ni, Cr, and Ti were tested as protective coatings between the thermoelectric material and liquid metal solder. W was recognized as a long-term stable coating material to protect materials from Ga-In-Sn solder. Although Cr and Ni react with Galinstan, they showed promising results as effective protective coatings for short-term applications. Reaction conditions and products are summarized in Table 5.

One of the problems for Ga in coolant applications is the serious corrosion that occurs when Ga comes into contact with Al alloys, which are the construction materials most commonly used in nuclear power plant cooling systems. Several studies have focused on the reaction mechanisms of Ga and Ga-based alloys with the candidate structural materials in nuclear power plant cooling systems. Therefore, we could gain some knowledge about the reactions between liquid Ga-based alloys and other substrates from these corrosion observations, as summarized in Table 5.

For atomic reactor cooling purposes, Fe, Ni, and Cr react with Ga quickly, whereas Nb_5_Mo_1_Zr alloy and 316 L stainless steel have better resistance to corrosion by Ga [107,108]. Further mathematical analysis of liquid Ga and liquid Ga-Sn-Zn alloy corrosion of austenitic stainless steels has been carried out [109]. The compatibility of Ga with four typical substrates, including two Al alloys, a Cu alloy, and a stainless steel, was observed in the temperature range relevant to the cooling of computer chips [110].

### 4.2. Wettability

Wettability properties are vital for liquid alloys in nearly all applications, including interconnection fabrication. Taking soldering as an example, the success of the joining process depends on the development of a liquid interlayer that has the ability to wet the substrate materials. Factors identified as influencing wetting include liquid metal oxidation behavior, liquid-solid alloying, or corrosion reactions with substrates, and the substrate surface conditions including roughness, contamination, segregation, and oxides [111].

The wetting behavior of room temperature liquid Ga-based alloys on metal substrates, based on microelectronic interconnect applications, have not been well observed. In other applications, such as three-dimensional (3D) structure patterning, microfluidic and nano-particle fabrication, extensive research has been completed to study the wettability of liquid Ga-based alloys in order to maintain the required geometries. The studies include surface tension of alloy droplets in different atmosphere [112] and the substrate conditions [113,114]. Here, we briefly discuss the progress of wettability characterisation of Ga-based alloys on substrates.

#### 4.2.1. Wetting Characteristics of Liquid Ga and Ga-Based Alloys

The surface tension of pure Ga and its temperature dependence have been measured by the sessile drop technique [80,115] and the pendant-drop method [116].

For Galinstan and eutectic Ga-In, several measurements have been recorded at room temperature in different atmospheres. Liu et al. [82] obtained the contact angle of Galinstan on several materials commonly used in micro-electromechanical systems devices (including tungsten, silicon nitride, glass, parylene, Teflon, phlogopite, and muscovite) using the sessile drop method. The surface tension of Galinstan on these substrates was measured using the pendant-drop method under a nitrogen atmosphere. There were no chemical reactions between any of the substrates tested and Galinstan, and the surface tension of Galinstan was measured to be around 534.6 mN/m. The authors found that the oxidation state had an obvious effect on the Galinstan drop morphology and fluidity. During the wettability tests, only when the oxygen level was precisely controlled to be below 1 ppm, would Galinstan droplets behave like a normal liquid. The results confirmed the effect of the oxide skin on the Galinstan wettability on solid substrates. Xu et al. [117] measured the wettability including viscosity, surface tension, and contact angles of pure Ga and eutectic Ga-In alloy, while controlling the oxidation level by immersing the liquid metal drops in a hydrochloric acid bath with different HCl concentrations. The dynamic and static contact angles of eutectic Ga-In alloy on cellulose paper and double-sided adhesive were also obtained by Han et al. [85]. All these measurements were taken on substrates that do not chemically react with or are penetrated by these liquid Ga-based alloys.

As well as these wettability measurements at room temperature, the surface tension of liquid Ga-Bi, Ga-In, Ga-Bi-Sn, and Ga-Bi-In alloys at 600 °C were also measured using the sessile drop method on graphite substrates in a hydrogen atmosphere [118].

#### 4.2.2. Effect of Liquid-Solid Interaction on Wettability

For the Ga/solid thin film couples, interdiffusion was found to occur heterogeneously over the film surface, and blisters formed due to the diffusion-induced stresses [105].

The spreading and penetration phenomenon between Ga and thin film Ag was investigated at temperatures ranging from −78 to 60 °C [119]. The activation energies of solid and liquid Ga in linear spreading on silver films were obtained. The effects of IMCs or solid solution formation, grain boundary diffusion, and grooving were examined, and models were produced for the spreading rates. This kind of investigation and associated mathematical models may be helpful in many liquid metal applications, including, for example, low temperature soldering.

## 5. Summary

The application of Ga and Ga-based alloys in microelectronics has already received significant attention. The most attractive properties of Ga and Ga-based alloys arise from their combination of thermal and electrical conductivity, with fluidity at low temperatures or even at room temperature. In addition, they exhibit other desirable properties, such as low toxicity, an ability to wet almost all materials used in electronics, high boiling points, low vapour pressure, and the possibility of forming stable high temperature solid solutions and IMCs with other metals (Al, Cu, Ni, etc.).

The properties of Ga and Ga-based alloys make them very suitable for use in microelectronics. Their deformability creates opportunities for further applications, providing a potential pathway to meet the demands for deformability and miniaturisation of products, low fabrication temperatures, and minimising the impact on both human health and the environment. Demonstrations of Ga and liquid Ga-based alloy applications in microelectronics have been reported in the literature, including deformable electronics fabrication, thermal management or heat transfer in integrated circuit systems, and low temperature bonding in electronic packaging.

Various characteristics of Ga and Ga-based alloys (mainly eutectic Ga-In and Galinstan) have been observed. This research has laid the foundation for some techniques for liquid alloy control and utilisation. For example, methods for “printing” or “writing” liquid Ga metals is emerging as a popular research area. However, there are relatively few studies relating to the interfacial reactions between liquid Ga-based alloys and common substrates in microelectronics.

Though Ga and Ga-based alloys hold some appeal for the electronics industry, a fundamental understanding about the interfacial reactions between liquid Ga-based alloys and other components and substrates at low temperatures needs to be developed to fully utilise these interesting materials and expedite their use in industry. Their long-term reliability in real devices where they have been used for low temperature bonding or their stretchability remains to be verified.

## Figures and Tables

**Table 1:** Physical properties of gallium (Ga), Ga-based alloys, and mercury (Hg) [5,6,7,8,10,11,12].

Parameter	Ga	EGaIn	Galinstan	Hg
Melting point (°C)	29.76	15.5	−19.0	−38.8
Boiling point Tb (°C)	2403	2000	>1300	356
Density at 20 °C (g/cm^3^)	5.90	6.280	6.440	13.533
Vapour pressure (Pa)	1 at 1037 °C	<1.33 × 10^−10^ at 300 °C	<1.33 × 10^−6^ at 500 °C	1 at 42 °C
Specific heat (J/kg/K)	410	404	295	140
Electrical conductivity (W/m/K)	6.73 × 10^6^	3.40 × 10^6^	3.46 × 10^6^	1.04 × 10^6^
Thermal conductivity (W/m/K)	29.3	26.6	16.5	8.5
Viscosity μ (kg/m/s)	1.37 × 10^−3^	1.99 × 10^−3^	2.4 × 10^−3^	1.526 × 10^−3^

**Table 2:** Summary of reactions between liquid Ga-based alloys and metal powders.

Liquid Phase	Powder Phase	Products	Reference
Ga ^1^	Cu	CuGa_2_ + Cu	[93]
Ga ^1^	Ni	NiGa_4_ + Ni	[93]
Ga ^1^	Cu-20% Ga Solid solution	CuGa_2_	[90]
Ga ^1^	Cu_9_Ga_4_	CuGa_2_	[90]
Ga-12% Sn	Cu	CuGa_2_ + Sn	[92]
Ga ^1^	Cu-20% Sn solid solution	CuGa_2_ + Sn (Ga) ^2^	[95]
Ga-12% Sn	Cu-20% Sn solid solution	CuGa_2_ + Sn	[91]
Ga-12% Sn	Cu-39% Sn (Cu_3_Sn)	CuGa_2_ + Sn (Ga) ^2^	[95]
Ga-12% Sn	Cu-61% Sn (Cu_6_Sn_5_)	CuGa_2_ + Sn (Ga) ^2^	[95]
Ga-24.5% In	Cu-20% In solid solution	CuGa_2_ + In	[91]
Ga-12% Sn	Cu-20% In solid solution	CuGa_2_ + In_3_Sn + Sn	[91]
Ga-24.5% In	Cu-20% Sn solid solution	CuGa_2_ + InSn_4_ + In	[91]
Ga ^1^	Cu-10% Bi solid solution	CuGa_2_ + Bi + Cu	[94]
Ga-24.5% In	Cu-10% Bi solid solution	CuGa_2_ + BiIn_2_	[91]
Ga-12% Sn	Cu-10% Bi solid solution	CuGa_2_ + Bi + In	[91]
Ga-19% In-16% Sn	Ag-25.7% Sn-15% Cu-9% Pd-0.3% Zn powder	Cu_9_Ga_4_ + Ag_9_In_4_ + Ag_2_Ga + Cu (Pd)Ga_2_ + Ga_28_Ag_72_ + Sn + Ag_3_Sn	[97,98,99]

All the reactions were analysed at room temperature (~20 °C). Compositions are in weight percent; ^1^ Pure Ga was brought to a liquid state at ~37 °C before the mixture; ^2^ Sn(Ga) stands for Ga solid solution in Sn.

**Table 3:** Summary of reactions between liquid Ga-based alloys and Cu substrates.

Liquid Phase	Solid Phase	Reaction Condition		Products	Reference
		Temperature	Time		
Ga	Cu foil	200 °C	3–24 h	CuGa_2_	[59]
Ga	Cu	160–240 °C	3–48 h	CuGa_2_ Cu_9_Ga_4_	[60]
Ga	Cu	280–300 °C	3–48 h	Cu_9_Ga_4_	[60]
Ga on a 50 nm Au seed layer	Cu	25 °C	10 min	CuGa_2_ Cu_9_Ga_4_ AuGa_2_	[61]
		50 or 90 °C	80 h		
Ga on a 50 nm Au seed layer	Cu	25 °C	10 min	Cu_9_Ga_4_ AuGa_2_	[61]
		200 °C	80 h		
Sn-32% Bi-6% Ga	Cu	158 °C	1–8 min	CuGa_2_	[62]
		70–110 °C	0–720 h		
Ga-10% Zn	Cu	150 or 200 °C	Not mentioned	Cu_9_Ga_4_ Zn	[63]
Ga	Two Cu foil	160 °C	96 h	CuGa_2_ Cu_9_Ga_4_	[60]
Ga	Two Cu foil coated with 40 nm thick Pt, Ga/Pt thickness ≤ 1	300 °C	7 h	Ga_7_Pt_3_	[59]
Ga	Two Cu foil coated with 40 nm thick Pt, Ga/Pt thickness ≥ 4	300 °C	7 h	Cu_9_Ga_4_ Cu (Ga) Ga_7_Pt_3_	[59]
Ga-13.5% Sn	Two Au coated Cu foil	25 or 100 °C	7 days	CuGa_2_	[64]

Compositions are in weight percent.

**Table 4:** Volume changes associated with interfacial reactions in soldering process [60].

Reaction	Volume Shrinkage
$3\mathrm{Ag}+\mathrm{Sn}={\mathrm{Ag}}_{3}\mathrm{Sn}$	6.01%
$9\mathrm{Ag}+4\mathrm{In}={\mathrm{Ag}}_{9}{\mathrm{In}}_{4}$	−12.4% (expansion)
$3\mathrm{Cu}+\mathrm{Sn}={\mathrm{Cu}}_{3}\mathrm{Sn}$	8.69%
$6\mathrm{Cu}+5\mathrm{Sn}={\mathrm{Cu}}_{6}{\mathrm{Sn}}_{5}$	6.83%
$\mathrm{Cu}+2\mathrm{Ga}={\mathrm{CuGa}}_{2}$	0.46%
$9\mathrm{Cu}+4\mathrm{Ga}={\mathrm{Cu}}_{9}{\mathrm{Ga}}_{4}$	6.26%

**Table 5:** Summary of reactions between liquid Ga-based alloys and substrates other than Cu.

Liquid Phase	Solid Phase	Reaction Condition		Products	Reference
		Temperature	Time		
Ga	Ni	300 °C	24–3000 h	Ni_2_Ga_3_, NiGa_4_	[107]
Ga	Fe	300 °C	24–3000 h	FeGa_3_	[107]
Ga	Cr	300 °C	24–3000 h	CrGa_4_	[107]
Ga	Pd	~25 °C	8 days	PdGa_5_	[101]
Ga	Au	≤ 50 °C	10 min	AuGa_2_	[102]
Ga	Stainless steel 316 (Fe-17% Cr-13% Ni-2.5% Mo)	400 °C	24–3000 h	FeGa_3_, CrGa_4_, Ni_2_Ga_3_	[107]
Ga	Inconel 625 (Ni-21.5% Cr-9% Mo-2.5Fe)	400 °C	24–3000 h	CrGa_4_	[107]
Galinstan	Ni	500 °C	24 h	Ga_65_Ni_35_, In _50_Ga_25_Sn_20_Ni_5_, In_55_Sn_41_Ga_4_	[106]
Galinstan	Ti	500 °C	24 h	Ga_75_Ti_25_, Ga_72_In_12_Ti_9_Sn_7_	[106]
Galinstan	Cr	500 °C	24 h	Ga_70_In_13_Cr_9_Sn_8_, Cr_84_Ga_13_In_3_Sn_1_	[106]
Galinstan	W	500 °C	24 h	No reaction layer was found	[106]
Ga-10% Zn	Cu-37% Zn or Cu-32% Zn	150 or 200 °C	Not mentioned	Cu_9_Ga_4,_ Cu solid solution	[63]
Ga-45% Al	a Cu foil and a Ni foil	700 °C	20 min	Ni_3_Ga, Cu_3_Ga, Cu solid solution Cu_9_Ga_4_	[66]
Ga-40% Ni-15% Al	a Cu foil and a Ni foil	700 °C	20 min	Ni_3_Ga, Cu_3_Ga, Cu solid solution	[66]

Compositions are in weight percent.

## References

[1] Waldrop M.M. (2016). The chips are down for Moore’s law. Nat. News.

[2] Hsiao H.Y., Liu C.M., Lin H., Liu T.C., Lu C.L., Huang Y.S., Chen C., Tu K.N. (2012). Unidirectional Growth of Microbumps on (111)-Oriented and Nanotwinned Copper. Science.

[3] Obama B. (2017). The irreversible momentum of clean energy. Science.

[4] Directive (EU) 2017/2102 of the European Parliament and of the Council of 15 November 2017 Amending Directive 2011/65/EU on the Restriction of the Use of Certain Hazardous Substances in Electrical and Electronic Equipment (Text with EEA Relevance.). https://eur-lex.europa.eu/legal-content/EN/TXT/?uri=CELEX%3A32017L2102.

[5] Martienssen W. (2005). The Elements. Springer Handbook of Condensed Matter and Materials Data.

[6] Greber J.F. (2000). Gallium and Gallium Compounds.

[7] Dickey M.D., Chiechi R.C., Larsen R.J., Weiss E.A., Weitz D.A., Whitesides G.M. (2008). Eutectic Gallium-Indium (EGaIn): A Liquid Metal Alloy for the Formation of Stable Structures in Microchannels at Room Temperature. Adv. Funct. Mater..

[8] Geratherm Medical AG Galinstan Safety Data Sheet. http://www.rgmd.com/msds/msds.pdf.

[9] Rumble J. (2017). CRC Handbook of Chemistry and Physics.

[10] Prokhorenko V.Y., Roshchupkin V.V., Pokrasin M.A., Prokhorenko S.V., Kotov V.V. (2000). Liquid Gallium: Potential Uses as a Heat-Transfer Agent. High Temp..

[11] Yang X.H., Tan S.C., Liu J. (2016). Numerical investigation of the phase change process of low melting point metal. Int. J. Heat Mass Transf..

[12] Hunter W.R., Williams R.T. (1984). Grain boundary diffusion of liquid metal coolants in optical materials for use with high power synchrotron radiation. Nucl. Instrum. Methods Phys. Res..

[13] Perepezko J.H. (1984). Nucleation in undercooled liquids. Mater. Sci. Eng..

[14] Zhu S., So J.-H., Mays R., Desai S., Barnes W.R., Pourdeyhimi B., Dickey M.D. (2013). Ultrastretchable Fibers with Metallic Conductivity Using a Liquid Metal Alloy Core. Adv. Funct. Mater..

[15] USGS Minerals Information: Gallium. https://minerals.usgs.gov/minerals/pubs/commodity/gallium/.

[16] Naumov A.V. (2013). Status and prospects of world gallium production and the gallium market. Metallurgist.

[17] (2014). Gallium: Global Industry Markets & Outlook.

[18] Puttkammer A. (1928). Mercury-free amalgam. Zahnaerztl. Rundsch..

[19] Smith D.L., Caul H.J. (1956). Alloys of gallium with powdered metals as possible replacement for dental amalgam. J. Am. Dent. Assoc..

[20] Shaker R.E., Brantley W.A., Wu Q., Culbertson B.M. (2001). Use of DSC for study of the complex setting reaction and microstructural stability of a gallium-based dental alloy. Thermochim. Acta.

[21] J.S.H. (1926). Gallium in quartz thermometer. J. Frankl. Inst..

[22] Lefrant J.Y., Muller L., de Coussaye J.E.L., Benbabaali M., Lebris C., Zeitoun N., Mari C., Saïssi G., Ripart J., Eledjam J.J. (2003). Temperature measurement in intensive care patients: Comparison of urinary bladder, oesophageal, rectal, axillary, and inguinal methods versus pulmonary artery core method. Intensive Care Med..

[23] Rubia-Rubia J., Arias A., Sierra A., Aguirre-Jaime A. (2011). Measurement of body temperature in adult patients: Comparative study of accuracy, reliability and validity of different devices. Int. J. Nurs. Stud..

[24] Speckbrock G., Kamitz S., Alt M., Schmitt H. (2000). Low Melting Gallium, Indium, and Tin Eutectic Alloys, and Thermometers Employing Same. USA Patent.

[25] Sawada T., Netchaev A., Ninokata H., Endo H. (2000). Gallium-cooled liquid metallic-fueled fast reactor. Prog. Nucl. Energy.

[26] Buligins L., Thomsen K., Lielausis O., Platacis E., Poznaks A. (2014). Internal geometry and coolant choices for solid high power neutron spallation targets. Nucl. Instrum. Methods Phys. Res. Sect. Accel. Spectrom. Detect. Assoc. Equip..

[27] Jung J.A., Kim S.H., Shin S.H., Bang I.C., Kim J.H. (2013). Feasibility study of fuel cladding performance for application in ultra-long cycle fast reactor. J. Nucl. Mater..

[28] Lee S.W., Park S.D., Kang S., Shin S.H., Kim J.H., Bang I.C. (2012). Feasibility study on molten gallium with suspended nanoparticles for nuclear coolant applications. Nucl. Eng. Des..

[29] Sharma D., Singh P.P., Garg H. (2013). Comparative Study of Rectangular and Trapezoidal Microchannels Using Water and Liquid Metal. Procedia Eng..

[30] Ge H., Liu J. (2013). Keeping Smartphones Cool with Gallium Phase Change Material. J. Heat Transf..

[31] Ge H., Liu J. (2013). Cooling Capacity of Metal Phase Change Material for Thermal Management of Mobile Phone Subject to Long Time Communication. ASME 2013 International Mechanical Engineering Congress and Exposition.

[32] Deng Y., Liu J. (2010). Design of Practical Liquid Metal Cooling Device for Heat Dissipation of High Performance CPUs. J. Electron. Packag..

[33] Deng Y., Liu J. (2013). Optimization and Evaluation of a High-Performance Liquid Metal CPU Cooling Product. IEEE Trans. Compon. Packag. Manuf. Technol..

[34] Zhu J.Y., Tang S.Y., Khoshmanesh K., Ghorbani K. (2016). An Integrated Liquid Cooling System Based on Galinstan Liquid Metal Droplets. ACS Appl. Mater. Interfaces.

[35] Ma K.Q., Liu J. (2007). Heat-driven liquid metal cooling device for the thermal management of a computer chip. J. Phys. Appl. Phys..

[36] Dickey M.D. (2017). Stretchable and Soft Electronics using Liquid Metals. Adv. Mater..

[37] Khoshmanesh K., Tang S.Y., Yang Zhu J., Schaefer S., Mitchell A., Kalantar-zadeh K., Dickey M.D. (2017). Liquid metal enabled microfluidics. Lab. Chip.

[38] Blaiszik B.J., Kramer S.L.B., Grady M.E., McIlroy D.A., Moore J.S., Sottos N.R., White S.R. (2012). Autonomic Restoration of Electrical Conductivity. Adv. Mater..

[39] Mineart K.P., Lin Y., Desai S.C., Krishnan A.S., Spontak R.J., Dickey M.D. (2013). Ultrastretchable, cyclable and recyclable 1- and 2-dimensional conductors based on physically cross-linked thermoplastic elastomer gels. Soft Matter.

[40] Palleau E., Reece S., Desai S.C., Smith M.E., Dickey M.D. (2013). Self-Healing Stretchable Wires for Reconfigurable Circuit Wiring and 3D Microfluidics. Adv. Mater..

[41] Kawakami H. (2008). Polymeric membrane materials for artificial organs. J. Artif. Organs.

[42] Baughman R.H. (2005). Playing Nature’s Game with Artificial Muscles. Science.

[43] Gao M., Gui L. (2016). Development of a fast thermal response microfluidic system using liquid metal. J. Micromech. Microeng..

[44] Gao Y., Bando Y. (2002). Nanotechnology: Carbon nanothermometer containing gallium. Nature.

[45] Gao Y., Bando Y. (2002). Nanothermodynamic analysis of surface effect on expansion characteristics of Ga in carbon nanotubes. Appl. Phys. Lett..

[46] Sivan V., Tang S.Y., O’Mullane A.P., Petersen P., Eshtiaghi N., Kalantar-zadeh K., Mitchell A. (2013). Liquid Metal Marbles. Adv. Funct. Mater..

[47] Shafiei M., Motta N., Hoshyargar F., O’Mullanc A.P. Development of new gas sensors based on oxidized galinstan. Proceedings of the 2015 IEEE SENSORS.

[48] Kim B., Jang J., You I., Park J., Shin S., Jeon G., Kim J.K., Jeong U. (2015). Interfacing Liquid Metals with Stretchable Metal Conductors. ACS Appl. Mater. Interfaces.

[49] Jeong Y.R., Kim J., Xie Z., Xue Y., Won S.M., Lee G., Jin S.W., Hong S.Y., Feng X., Huang Y. (2017). A skin-attachable, stretchable integrated system based on liquid GaInSn for wireless human motion monitoring with multi-site sensing capabilities. NPG Asia Mater..

[50] Krupenkin T., Taylor J.A. (2011). Reverse electrowetting as a new approach to high-power energy harvesting. Nat. Commun..

[51] Jeong S.H., Hjort K., Wu Z. (2015). Tape Transfer Atomization Patterning of Liquid Alloys for Microfluidic Stretchable Wireless Power Transfer. Sci. Rep..

[52] Joshipura I.D., Ayers H.R., Majidi C., Dickey M.D. (2015). Methods to pattern liquid metals. J. Mater. Chem. C.

[53] Tabatabai A., Fassler A., Usiak C., Majidi C. (2013). Liquid-Phase Gallium–Indium Alloy Electronics with Microcontact Printing. Langmuir.

[54] Lazarus N., Bedair S.S., Kierzewski I.M. (2017). Ultrafine Pitch Stencil Printing of Liquid Metal Alloys. ACS Appl. Mater. Interfaces.

[55] Daalkhaijav U., Yirmibesoglu O.D., Walker S., Mengüç Y. (2018). Rheological Modification of Liquid Metal for Additive Manufacturing of Stretchable Electronics. Adv. Mater. Technol..

[56] Ladd C., So J.H., Muth J., Dickey M.D. (2013). 3D Printing of Free Standing Liquid Metal Microstructures. Adv. Mater..

[57] Zheng Y., He Z.Z., Yang J., Liu J. (2014). Personal electronics printing via tapping mode composite liquid metal ink delivery and adhesion mechanism. Sci. Rep..

[58] Kotadia H.R., Howes P.D., Mannan S.H. (2014). A review: On the development of low melting temperature Pb-free solders. Microelectron. Reliab..

[59] Lin S., Chang H., Cho C., Liu Y., Kuo Y. (2015). Formation of solid-solution Cu-to-Cu joints using Ga solder and Pt under bump metallurgy for three-dimensional integrated circuits. Electron. Mater. Lett..

[60] Lin S., Cho C., Chang H. (2013). Interfacial Reactions in Cu/Ga and Cu/Ga/Cu Couples. J. Electron. Mater..

[61] Froemel J., Baum M., Wiemer M., Gessner T. (2015). Low-Temperature Wafer Bonding Using Solid-Liquid Inter-Diffusion Mechanism. J. Microelectromech. Syst..

[62] Chen C.H., Lee B.H., Chen H.C., Wang C.M., Wu A.T. (2015). Interfacial Reactions of Low-Melting Sn-Bi-Ga Solder Alloy on Cu Substrate. J. Electron. Mater..

[63] Mikheev A.A., Temnykh V.I., Kazakov V.S., Temnykh E.V., Mityaev A.E., Zeer G.M., Abkaryan A.K. (2012). Kinetics and products of interaction of zinc-containing gallium pastes–solders. Weld. Int..

[64] Liu S.Q., Qu D.D., McDonald S.D., Nogita K. (2018). The Interaction of Sn-Ga Alloys and Au Coated Cu Substrates. Solid State Phenom..

[65] Temnykh V.I., Kazakov V.S., Mityaev A.E., Temnykh E.V. (2012). Composite gallium soldering pastes for low-temperature diffusion soldering of cermet sections. Weld. Int..

[66] Sommadossi S., Troiani H.E., Guillermet A.F. (2007). Diffusion soldering using a Gallium metallic paste as solder alloy: Study of the phase formation systematics. J. Mater. Sci..

[67] Baldwin D.F., Deshmukh R.D., Hau C.S. (2000). Gallium alloy interconnects for flip-chip assembly applications. IEEE Trans. Compon. Packag. Technol..

[68] Bhattacharya S.K., Baldwin D.F. (2000). A low temperature processable ternary gallium alloy for via filling application in microelectronic packaging. J. Mater. Sci. Mater. Electron..

[69] Çınar S., Tevis I.D., Chen J., Thuo M. (2016). Mechanical Fracturing of Core-Shell Undercooled Metal Particles for Heat-Free Soldering. Sci. Rep..

[70] Stagon S., Knapp A., Elliott P., Huang H. (2016). Metallic glue for ambient environments making strides. Adv. Mater. Process..

[71] Grigor’eva T.F., Kovaleva S.A., Barinova A.P., Šepelák V., Vityaz’ P.A., Lyakhov N.Z. (2011). Properties of metallic cements formed upon the interaction of mechanochemically synthesized copper alloys with liquid gallium and its eutectics: Interaction of Cu/Bi composites with liquid gallium. Phys. Met. Metallogr..

[72] Ye Z., Lum G.Z., Song S., Rich S., Sitti M. (2016). Phase Change of Gallium Enables Highly Reversible and Switchable Adhesion. Adv. Mater..

[73] Gao Y., Bando Y., Liu Z., Golberg D., Nakanishi H. (2003). Temperature measurement using a gallium-filled carbon nanotube nanothermometer. Appl. Phys. Lett..

[74] Zhang R., Hodes M., Lower N., Wilcoxon R. (2015). Water-Based Microchannel and Galinstan-Based Minichannel Cooling Beyond 1 kW/cm^2^ Heat Flux. IEEE Trans. Compon. Packag. Manuf. Technol..

[75] Khan M.R., Trlica C., Dickey M.D. (2015). Recapillarity: Electrochemically Controlled Capillary Withdrawal of a Liquid Metal Alloy from Microchannels. Adv. Funct. Mater..

[76] Khan M.R., Eaker C.B., Bowden E.F., Dickey M.D. (2014). Giant and switchable surface activity of liquid metal via surface oxidation. Proc. Natl. Acad. Sci. USA.

[77] Tang S.Y., Sivan V., Khoshmanesh K., O’Mullane A.P., Tang X., Gol B., Eshtiaghi N., Lieder F., Petersen P., Mitchell A. (2013). Electrochemically induced actuation of liquid metal marbles. Nanoscale.

[78] Wang L., Liu J. (2015). Electromagnetic rotation of a liquid metal sphere or pool within a solution. Proc. R. Soc. Lond. Math. Phys. Eng. Sci..

[79] Xiong M., Gao Y., Liu J. (2014). Fabrication of magnetic nano liquid metal fluid through loading of Ni nanoparticles into gallium or its alloy. J. Magn. Magn. Mater..

[80] Chentsov V.P., Shevchenko V.G., Mozgovoi A.G., Pokrasin M.A. (2011). Density and surface tension of heavy liquid-metal coolants: Gallium and indium. Inorg. Mater. Appl. Res..

[81] Liu T., Sen P., Kim C.J. (2012). Characterization of Nontoxic Liquid-Metal Alloy Galinstan for Applications in Microdevices. J. Microelectromech. Syst..

[82] Liu T., Sen P., Kim C.J. Characterization of liquid-metal Galinstan for droplet applications. Proceedings of the 2010 IEEE 23rd International Conference on Micro Electro Mechanical Systems (MEMS).

[83] Mohammed M., Sundaresan R., Dickey M.D. (2015). Self-Running Liquid Metal Drops that Delaminate Metal Films at Record Velocities. ACS Appl. Mater. Interfaces.

[84] Zhang J., Yao Y., Sheng L., Liu J. (2015). Self-Fueled Biomimetic Liquid Metal Mollusk. Adv. Mater..

[85] Long Han Y., Liu H., Ouyang C., Jian Lu T., Xu F. (2015). Liquid on Paper: Rapid Prototyping of Soft Functional Components for Paper Electronics. Sci. Rep..

[86] Wang L., Liu J. (2015). Pressured liquid metal screen printing for rapid manufacture of high resolution electronic patterns. RSC Adv..

[87] Mohammed M.G., Xenakis A., Dickey M.D. (2014). Production of Liquid Metal Spheres by Molding. Metals.

[88] Tang S.Y., Ayan B., Nama N., Bian Y., Lata J.P., Guo X., Huang T.J. (2016). On-Chip Production of Size-Controllable Liquid Metal Microdroplets Using Acoustic Waves. Small.

[89] Tang S.Y., Joshipura I.D., Lin Y., Kalantar-Zadeh K., Mitchell A., Khoshmanesh K., Dickey M.D. (2016). Liquid-Metal Microdroplets Formed Dynamically with Electrical Control of Size and Rate. Adv. Mater..

[90] Ancharov A.I., Grigoryeva T.F., Barinova A.P., Boldyrev V.V. (2009). Interaction between copper and gallium. Russ. Metall. Met..

[91] Ancharov A.I., Grigorieva T.F., Tsybulya S.V., Boldyrev V.V. (2006). Chemical interaction of Cu-In, Cu-Sn, and Cu-Bi solid solutions with liquid Ga-In and Ga-Sn eutectics. Inorg. Mater..

[92] Ancharov A.I., Grigoriyeva T.F., Tsybulya S.V., Boldyrev V.V. (2006). Interaction of copper-based solid solutions with liquid gallium eutectics. Russ. Metall. Met..

[93] Grigoreva T.F., Ancharov A.I., Barinova A.P., Tsybulya S.V., Lyakhov N.Z. (2009). Structural transformations upon the mechanochemical interaction between solid and liquid metals. Phys. Met. Metallogr..

[94] Grigor’eva T.F., Ancharov A.I., Kovaleva S.A., Barinova A.P., Becker K.D., Šepelák V., Lyakhov N.Z. (2010). Study of the chemical interaction between mechanochemically synthesized Cu/Bi nanocomposites and liquid gallium. Russ. J. Appl. Chem..

[95] Grigor’eva T.F., Ancharov A.I., Manzyrykchy K.B., Becker K.D., Šepelak V., Barinova A.P., Lyakhov N.Z. (2010). How the tin concentration affects the interactions of intermetallic compounds of the Cu-Sn system with liquid gallium and a gallium-tin eutectic. Russ. J. Inorg. Chem..

[96] Grigor’eva T.F., Ancharov A.I., Barinova A.P., Tsybulya S.V., Lyakhov N.Z. (2009). Structural transformations in mechanochemical synthesis of solid solutions in the Cu-Ga system. Russ. J. Appl. Chem..

[97] Herø H., Simensen C.J., Jørgensen R.B. (1996). Structure of dental gallium alloys. Biomaterials.

[98] Gunnæs A.E., Olsen A., Herø H. (1996). Transmission electron microscopy study of a dental gallium alloy. J. Mater. Sci. Mater. Med..

[99] Hero H., Okabe T. (1994). Gallium alloys as dental restorative materials: A research review. Cells Mater..

[100] Weibke F., Villars P., Okamoto H., Cenzual K. (2016). Cu-Ga Phase Diagram, ASM Alloy Phase Diagrams Database.

[101] Marinković Ž., Simić V. (1992). Comparative analysis of interdiffusion in some thin film metal couples at room temperature. Thin Solid Films.

[102] Frömel J., Lin Y.C., Wiemer M., Gessner T., Esashi M. Low temperature metal interdiffusion bonding for micro devices. Proceedings of the 2012 3rd IEEE International Workshop on Low Temperature Bonding for 3D Integration.

[103] Tang J., Zhao X., Li J., Guo R., Zhou Y., Liu J. (2017). Gallium-Based Liquid Metal Amalgams: Transitional-State Metallic Mixtures (TransM2ixes) with Enhanced and Tunable Electrical, Thermal, and Mechanical Properties. ACS Appl. Mater. Interfaces.

[104] Kulikova T.V., Bykov V.A., Shunyaev K.Y., Shubin A.B. (2012). Thermal Properties of CuGa2 Phase in Inert Atmosphere. Defect Diffus. Forum.

[105] Nakahara S., Kinsbron E. (1984). Room temperature interdiffusion study of Au/Ga thin film couples. Thin Solid Films.

[106] Kolb H., Sottong R., Dasgupta T., Mueller E., de Boor J. (2017). Evaluation of Detachable Ga-Based Solder Contacts for Thermoelectric Materials. J. Electron. Mater..

[107] Luebbers P.R., Chopra O.K. Compatibility of ITER candidate materials with static gallium. Proceedings of the 16th International Symposium on Fusion Engineering.

[108] Narh K.A., Dwivedi V.P., Grow J.M., Stana A., Shih W.-Y. (1998). The effect of liquid gallium on the strengths of stainless steel and thermoplastics. J. Mater. Sci..

[109] Shin S.H., Kim S.H., Kim J.H. (2014). Model of liquid gallium corrosion with austenitic stainless steel at a high temperature. J. Nucl. Mater..

[110] Deng Y.G., Liu J. (2009). Corrosion development between liquid gallium and four typical metal substrates used in chip cooling device. Appl. Phys. A.

[111] Gale W.F., Butts D.A. (2004). Transient liquid phase bonding. Sci. Technol. Weld. Join..

[112] Zhao X., Xu S., Liu J. (2017). Surface tension of liquid metal: Role, mechanism and application. Front. Energy.

[113] Yoon Y., Kim D., Lee J.-B. (2014). Hierarchical micro/nano structures for super-hydrophobic surfaces and super-lyophobic surface against liquid metal. Micro Nano Syst. Lett..

[114] Kramer R.K., Boley J.W., Stone H.A., Weaver J.C., Wood R.J. (2014). Effect of Microtextured Surface Topography on the Wetting Behavior of Eutectic Gallium–Indium Alloys. Langmuir.

[115] Hardy S.C. (1985). The surface tension of liquid gallium. J. Cryst. Growth.

[116] Abbaschian G.J. (1975). Surface tension of liquid gallium. J. Less Common Met..

[117] Xu Q., Oudalov N., Guo Q., Jaeger H.M., Brown E. (2012). Effect of oxidation on the mechanical properties of liquid gallium and eutectic gallium-indium. Phys. Fluids.

[118] Tanaka T., Matsuda M., Nakao K., Katayama Y., Kaneko D., Hara S., Xing X., Qiao Z. (2001). Measurement of surface tension of liquid Ga-base alloys by a sessile drop method. Z. Für Met..

[119] Glickman E., Levenshtein M., Budic L., Eliaz N. (2011). Interaction of liquid and solid gallium with thin silver films: Synchronized spreading and penetration. Acta Mater..

